# Sulphate Morphia as a Parturient

**Published:** 1871-03

**Authors:** L. D. Robinson

**Affiliations:** Coatsville Ind.


### SULPHATE MORPHIA AS A PARTURIENT.

By L. D. ROBINSON, M.D., of Coatsville, Ind.

In January number of *Examiner*, I see the above subject briefly alluded to by Robert Robson, M.D., New Harmony, Ind. The Doctor says: "I have had frequent occasion to administer this article (morphia sulphas) in labor, in cases of nervous irritability, with a view to its anodyne effect, when it has resulted in producing effects similar to the secale cornutum."

With all due deference to the venerable Doctor's opinion on the *modus operandi* of sul. morphia, in such cases, I beg leave to respectfully dissent from his views as quoted above. I have, for several years, been accustomed to administer sul. morphia in all cases where there was irritability of nervous system, and the labor protracted by rigidity of the os uteri, or perineum. And in all, and every such case, it has not only given the patient great relief, but, in my judgment, very much accelerated the labor. Not, as I conceive, by directly, or otherwise, increasing uterine contraction, as secale cornutum does, *but, by its quieting and soothing influences upon the nervous system in general, including the uterine nerves, thus relaxing the rigid resisting parts, and permitting the natural physiological process of uterine contraction, and expulsion of fœtus, to go forward to completion of the process, more easily and rapidly.* When true labor sets in, it is not an abnormal process, but a strictly natural, physiological operation, and anodynes, nor even complete anæsthesia can, or will, for any considerable length of time, retard the process. But if there is any obstruction to the proper, natural process of parturition, from rigidity of the os uteri, or the perineum (which I regard as abnormal), either anodynes or anæsthesia will correct, and are the best means of correcting, this abnormal obstruction to the rapid termination of the labor.

Again, Dr. Robson says: "Such a property in the sulph. morphia would undoubtedly account for the many failures in the prevention of abortion where opiates have been given." My observation has been that morphia sulph. would prevent all cases of threatened abortion, where correctly administered, that were preventable; and I never knew it increase the uterine contractions in any case of abortion, or threatened abortion. I regard abortion, if not a diseased action, as unnatural and abnormal, and hence sulph. morphia, or anodynes, often completely control such cases, and they go on to natural labor.

Much more might be said on this interesting subject in this connection, but I presume I have said enough to be understood and I will close my remarks.

